# Highly stretchable and shape-controllable three-dimensional antenna fabricated by “Cut-Transfer-Release” method

**DOI:** 10.1038/srep42227

**Published:** 2017-02-13

**Authors:** Zhuocheng Yan, Taisong Pan, Guang Yao, Feiyi Liao, Zhenlong Huang, Hulin Zhang, Min Gao, Yin Zhang, Yuan Lin

**Affiliations:** 1 State Key Laboratory of Electronic Thin Films and Integrated Devices, University of Electronic Science and Technology of China, Chengdu, Sichuan 610054, P. R. China; 2 Center for Information in Medicine, University of Electronic Science and Technology of China, Chengdu, Sichuan 610054, P. R. China

## Abstract

Recent progresses on the Kirigami-inspired method provide a new idea to assemble three-dimensional (3D) functional structures with conventional materials by releasing the prestrained elastomeric substrates. In this paper, highly stretchable serpentine-like antenna is fabricated by a simple and quick “Cut-Transfer-Release” method for assembling stretchable 3D functional structures on an elastomeric substrate with a controlled shape. The mechanical reliability of the serpentine-like 3D stretchable antenna is evaluated by the finite element method and experiments. The antenna shows consistent radio frequency performance with center frequency at 5.6 GHz during stretching up to 200%. The 3D structure is also able to eliminate the hand effect observed commonly in the conventional antenna. This work is expected to spur the applications of novel 3D structures in the stretchable electronics.

With the outstanding performance in many fields such as healthcare monitoring^1^,^2^,^3^,^4^,^5^ and energy harvesting^6^,^7^, stretchable and flexible electronics have drawn much attention. To make them more convenient and comfortable for people, realizing wireless powering^8^ and communication^9^ in wearable electronics with the stretchable and flexible electronic devices have become popular topics. Antenna, as an essential part of a wireless system, is a key element in the design of stretchable and flexible electronic devices^9^. When a stretchable and flexible antenna is used in wearable electronics, its mechanical and radio-frequency reliability with stretching and bending during device operation become serious concerns for the performance of the device. To fabricate a highly stretchable antenna, some attempts on two-dimensional (2D) planar stretchable structures have been made with both novel materials and structural designs. These researches indicate that the radio frequency performance instability with large deformation of 2D planar structure prevents further improvement on the stretchability of an antenna^10^,^11^,^12^,^13^.

Instead of using simple 2D planar structures for an antenna, introducing 3D structures in antenna design is promising to provide a feasible solution for antennas used in wireless wearable electronics. However, the current 3D antenna researches focus mainly on the conventional unstretchable metal structures^14^,^15^, rather than structures with high stretchability. From perspective of fabrication techniques, the 3D printing technique has drawn much attention due to its convenience on building 3D structures in bottom-up way^16^,^17^,^18^,^19^. But the 3D printing technique also has some drawbacks. The materials suitable for 3D printing are limited. And it is difficult to prepare the additional functional thin film on the structures during the printing processes. Recently, a novel method proposed by Rogers *et al*.^20^,^21^,^22^,^23^ has offered a new route to assemble 3D structures in multiscale. In this Kirigami-inspired method, 2D precursor patterns can be transformed into 3D structures by releasing prestrained elastomer with the defined planner 2D precursor patterns bonded on. Although this method offers a more promising way to employ 3D structures in future stretchable electronics, its current fabrication technique makes it not an easy way to obtain 3D structures. The fabrication processes similar to conventional semiconductor technology, including photolithography and reactive ion etching, require high-cost equipments and a microelectronic standard clean room^24^. To promote applications of the 3D structures made by this top-down method, it is necessary to propose more solutions to simplify the fabrication and reduce costs.

Here, we report a method called “Cut-Transfer-Release” method for fabrication of highly stretchable 3D structures, which is expected to provide a simple and low-cost approach to realize transformation of 2D planar patterns into stretchable 3D structures. Instead of using the conventional lithography technique, the 2D precursor patterns are carved out by a low-cost programmable cutting machine first. The precursor patterns are then transferred to a prestrained elastomeric substrate only with a thermal release tape (TRT). The 2D to 3D transformation is completed by releasing the substrate and compressing of the 2D precursor. A 3D serpentine-like antenna is proposed and fabricated by this method, which exhibits mechanical and electrical robustness when the stretchability of the antenna is as high as 200%. The mechanical performance of the serpentine-like antenna structure is examined and compared with a ribbon structure by the finite element method (FEM) and experiments. The radio frequency properties of the serpentine-like antenna during stretching are measured and discussed.

## Results

**Figure 1:**
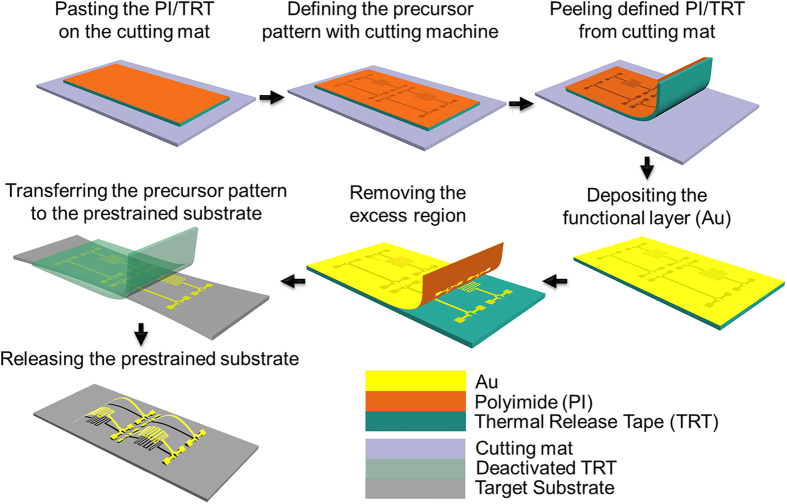
The fabrication process of the “Cut-Transfer-Release” method.

The process of the “Cut-Transfer-Release” method is illustrated in Fig. 1. To fabricate a 3D antenna by this method, the process starts from pasting a thermal release tape to a soft polymer film, for example, a 75-μm-thick polyimide (PI) in this work. The soft polymer film acts as a “framework” of the 3D structure. Both the thermal release tape and the 75-μm-thick PI film are commercially available. The other side of the thermal release tape is then adhered to a sticky flexible cutting mat. By using the commercial cutting machine (Silhouette Cameo, USA), the PI film on the cutting mat can be carved into a designed pattern. Once carving is completed, the patterned PI and thermal release tape are peeled from the cutting mat together. A functional thin film layer (50-nm-thick Au film in this work) is then deposited on the PI film. After baking the as-deposited sheet for 10 mins at 90 °C to reduce the adhesion between the TRT and the PI layer, the excess region of Au-covered PI layer can be removed. Then, the as-designed Au/PI pattern is left on the TRT. As the adhesion at the interface between the Au pattern and the TRT is lower than the one between Au and the target elastomeric substrate, the Au patterned sheet can be finally transferred onto the prestrained target elastomeric substrate (Ecoflex, Smooth-On, USA, Young’s modulus is 60 kPa). After the pre-defined bonding sites are bonded on the elastomeric substrate with Ecoflex gel (Smooth-On, USA), the transformation from 2D precursor pattern to 3D structure is accomplished by releasing the prestrained substrate (Fig. S1, Supplementary Information).

When the as-transformed 3D structure is stretched with an applied strain *ε*_*app*_ under working conditions, the length between two bonding sites, 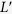. is dominated by the applied strain and the prestrain, which is given by


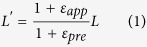


where *ε*_*pre*_ is the level of prestrain, *L* is the initial length between two bonding sites. The equivalent deformation can also be acquired by applying antoher specific prestrain 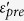 and then releasing. In this case, the corresponding prestrain 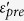 required to obtain the same 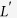
can be derived by


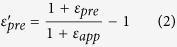


**Figure 2:**
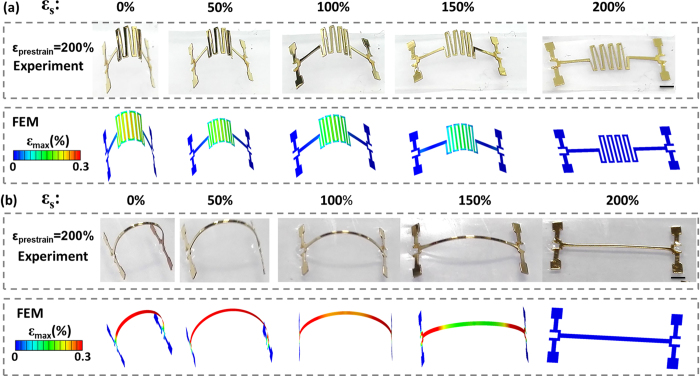
The optical images and FEM strain distribution results of 3D structures during releasing the 200% prestrained substrate. (**a**) the 3D Au/PI serpentine-like structure (**b**) the 3D Au/PI ribbon structure (scale bar: 1 mm).

As shown in the upper panel of Fig. 2(a), a serpentine pattern was used as the 2D precursor to assemble a 3D antenna first. The formation of the 3D antenna and detailed geometric dimensions can be seen in the Supplementary Information (Figs S2 and S3, Supplementary Information). The deformation states of the 3D antenna with the serpentine structure were characterized by optical imaging during releasing the 200% prestrained substrate. *ε*_s_ denotes the applied substrate strain. The optical image corresponding to *ε*_s_ = 0% indicates successful assembly of the 3D antenna when the elastomeric substrate was completely released (*ε*_s_ = 0%). The assembled 3D serpentine-like antenna was then stretched back to 200% (*ε*_s_ = 200%). The optical images prove that the entire structure did not break when the substrate strain *ε*_s_ increased back to as high as 200%. Further investigation on the strain distributions in Au layer of 3D antenna was conducted to examine the mechanical property for reliable stretchability performance. The strain distributions in the Au layer of the antenna with the serpentine structure under different substrate strain values (*ε*_s_), which are derived from the FEM results, are shown in the lower panel of Fig. 2(a). For the Au layer, a yield strain of 0.3% and a fracture strain of 5% are appropriate principles for the mechanical behavior of Au^25^. The elastic-plastic transition of Au is set when the maximum principal strain of half the width of one section is beyond the yield strain of 0.3%. The elastic-plastic transition plays a key role in determining the mechanical reliability of a deformed 3D structure. If the elastic-plastic transition happens in the Au layer, cracks will occur in the Au layer during multiple stretching loadings, which may induce mechanical failure to the antenna. According to the FEM results, obvious strain concentration can be observed in the straight segments of the serpentine structure after the prestrained substrate was fully released (*ε*_s_ = 0%). When the serpentine-like antenna was stretched to 200%, although the straight segments of the serpentine were still the strain concentrated regions, the strain in the Au layer kept decreasing with the stretching process. Moreover, even when *ε*_s_ = 0%, the maximum principal strain of half the width of any section in the Au layer was still smaller than 0.3% (no elastic-plastic transition happened), indicating the serpentine-like antenna exhibited good mechanical reliability. The bonding sites were also robust during the cyclic loading. (Fig. S4, Supplementary Information).

As shown in Fig. 2(b), a ribbon 2D precursor was also used to assemble a 3D structure. The optical images in the upper panel of Fig. 2(b) indicate that the 3D structure formed by releasing the 200% prestrained substrate with the ribbon 2D precursor. No breaking was observed in the optical images when this structure was stretched to *ε*_s_ =200% again. However, as observed in the strain distribution results obtained by FEM, which are shown in the lower panel of Fig. 2(b), the strain in Au layer for most areas of the ribbon were higher than 0.3% when the prestrained elastomeric substrate was fully released. It indicates that the elastic-plastic transition of Au layer has happened on the ribbon during releasing the prestrained elastomeric substrate, which has also been proved by the results of cyclic loading experiments (Fig. S5, Supplementary Information). The highly concentrated strain in the ribbon-like structure showed the significant strain distribution difference between the serpentine-like structure and the ribbon-like structure. The introduction of the serpentine structure can effectively improve the mechanical performance of a 3D antenna. During the transition from the 2D precursor pattern to the 3D structure, the serpentine structure acts like a stair and accomplishes the deformation with multiple straight segments instead of one segment in the ribbon structure. The increased number of straight segments offers better capacity for large deformation within a similar strain level. The serpentine structure, as highlighted by the comparison of FEM results of the serpentine structure and the ribbon structure, provides an opportunity to obtain a 3D structure using the simple “Cut-Transfer-Release” method while keeping the mechanical reliability.

**Figure 3:**
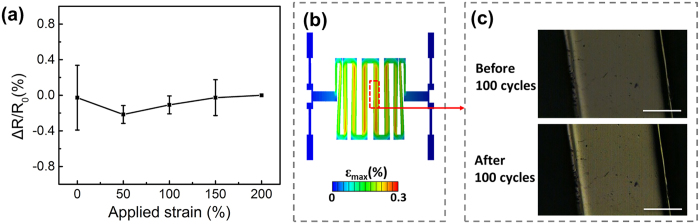
(**a**) The resistance change of serpentine-like antenna with different applied substrate strain after stretching to 200% for 100 times (the initial resistance R_0_ was measured when applied substrate strain is 200% and change value is average value of 10 samples); (**b**) The strain distribution derived from FEM result when the prestrained substrate is fully released; (**c**) Optical images of the strain concentration region before and after the substrate is stretched to 200% for 100 times (Scale bar: 100 um).

It has been reported that the electrical resistance of conductive thin film is closely correlated with crack density of the film^26^. When the strain in the Au thin film is high enough to induce the elastic-plastic transition during the deformation of the structure, cracks will appear in the Au thin film after multiple loadings. The existence of cracks will lead to obvious change of the electrical resistance of Au thin film. To study the reliability of the 3D antenna with the serpentine structure, the resistance change (ΔR) versus the applied substrate strain *ε*_s_ was measured after 100 cycles of stretching and compared with the initial resistance (R_0_) before stretching. The correlation between *ε*_s_ and ΔR/R_0_ is shown in Fig. 3(a). Only small resistance changes (less than 0.4% of the initial resistance R_0_) can be observed when the substrate was stretched with the applied strain in the range from 0% to 200%. The small ΔR/R_0_ value indicates that no obvious crack exists in the Au thin film after stretching of the serpentine structure, which is consistent with the FEM result of the strain distribution in the Au thin film layer. The mechanical reliability indicated by the resistance measurement is also confirmed by the optical imaging (Fig. S6, Supplementary Information). The high strain region in the FEM results shown in Fig. 3(b) was selected as the imaging region. The optical images of the selected region before and after cyclic loading are illustrated in Fig. 3(c). After the substrate was cyclically stretched from 0% to 200% for 100 times, no crack was observed on the serpentine-like 3D antenna. The cyclic loading test results provide experimental evidence that the serpentine structure, due to the low strain on the Au thin film, can survive from the large deformation during the cyclic stretching.

**Figure 4:**
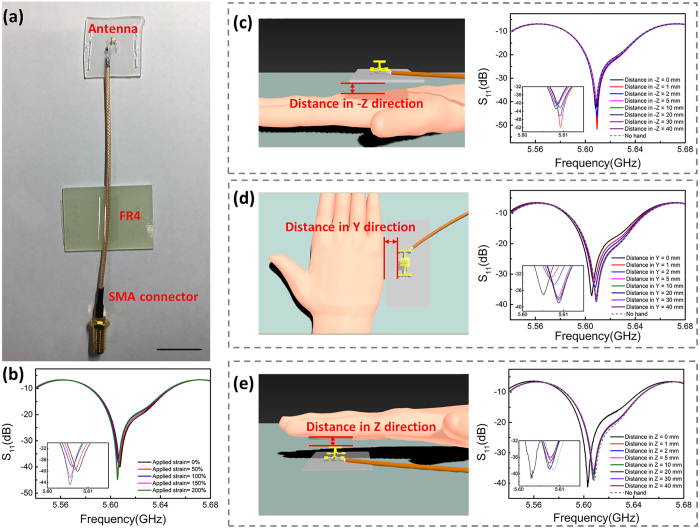
(**a**) Optical image of 3D serpentine-like antenna on Ecoflex with SMA connector and FR-4 substrate attached (Scale bar: 20 mm); (**b**) The S_11_ parameters of the 3D serpentine-like antenna with different applied substrate strain; (**c**) The S_11_ parameters of the 3D serpentine-like antenna with different distances to human hand in -Z direction; (**d**) The S_11_ parameters of the 3D serpentine-like antenna with different distances to human hand in Y direction; (**e**) The S_11_ parameters of the 3D serpentine-like antenna with different distances to human hand in Z direction.

The radio frequency (RF) performance of the 3D antenna with serpentine structure was evaluated by measuring the reflection coefficient (S_11_). The final assembly of the 3D serpentine-like antenna for measurement is shown in Fig. 4(a) more details in (Fig. S7, Supplementary Information). When an antenna is used in wearable electronics, it is very important to keep the stability of the operation frequency during the deformation of antenna. The S_11_ parameters of the serpentine-like 3D antenna with different substrate strains *ε*_s_ are plotted in Fig. 4(b). It can be observed that when the 3D serpentine-like antenna was stretched with *ε*_s_ from 0% to 200%, the S_11_ parameter kept lower than −10 dB from 5.59 GHz to 5.64 GHz, which is an assigned band for the 802.11a/h/j/n/ac wireless network. The radio frequency performance in 5.6 GHz band makes the 3D antenna with the serpentine structure a potential candidate for the antenna of high-speed wireless communication. The resonance frequency of the stretchable 3D antenna in this study can be calculated by 
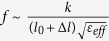
, where *f* is the resonance frequency, *l*_0_ is the initial length of antenna, Δl is the incremental length of antenna under uniaxial stretching, *ε*_*eff*_ is the effective dielectric constant of PI, and *k* is the correction factor^12^. According to this equation, the stable S11 parameter during stretching in Fig. 4(b) may be attributed to the structural difference between the 3D antennas we present here and the conventional planar stretchable antennas^27^,^28^,^29^,^30^. For conventional planar stretchable antennas, the large expansion and Poisson’s effect of stretched elastomeric substrate will significantly change the effective length of antenna (l0+Δl), so as to synchronously modulate the resonance frequency^12^. However, for the 3D structure formed by releasing prestrained substrate, the deformation relies on the out-of-plane mode. This kind of deformation only leads to marginally dimensional change rather than the length change of the serpentine structural unit, as indicated by the strain distribution derived from the FEM results. As the resonant characteristic mode is highly relevant to the overall size of antenna^31^, the RF performance of serpentine-like 3D antenna will not have obvious change with uniaxial stretching. With the reliable RF performance during deformation, the 3D antenna with a serpentine structure is expected to provide stable RF signal transmittal for the 802.11 high-speed wireless communications in wearable electronics.

As the human body will absorb the electromagnetic wave and have negative effects, for example, hand effect on the transmittal of wireless signals^32^, it is necessary to consider the influence of human body on the antenna RF performance when the antenna is used in wearable electronics. To simulate the RF performance in real application conditions, the S_11_ parameter of the 3D antenna with a serpentine structure was measured with different distances to a hand.

As shown in Fig. 4(c), the S_11_ parameter of the serpentine-like 3D antenna was firstly measured with a hand below to the antenna (-Z direction). The hand-antenna distance was 0 mm (the human hand directly contacted with the substrate), 1 mm, 2 mm, 5 mm, 10 mm, 20 mm, 30 mm and 40 mm, respectively. The measured S_11_ parameters in 5.6 GHz band in Fig. 4(c) illustrate that the resonance frequency remained stable when the hand-antenna distance changed from 0 mm to 40 mm. Similarly, as shown in Fig. 4(d), the S_11_ parameter of the serpentine-like 3D antenna was measured with a hand being parallel to the antenna (Y direction) with a hand-antenna distance changing from 0 mm to 40 mm. The plotted S_11_ parameters in Fig. 4(d) indicate that when the hand was approaching the antenna, resonance frequency shift to the negative side could be observed. Nevertheless, when comparing the S_11_ parameters for the case of no hand and that with a 10-mm-distance in Y-direction, the shift is less than 1% (from 5.6091 GHz to 5.6086 GHz). The S_11_ parameter of the antenna was also measured when a hand was on the top of it (Z direction). The relation between the frequency and the S_11_ parameter with a hand-antenna distance from 0 mm to 40 mm in the Z direction was plotted in Fig. 4(e). It can be observed that when the hand was far enough (the distance in the Z direction was larger than 4 cm), there was no significant hand effect for the 3D antenna with the serpentine structure, which was similar with the Y-direction case. When the hand moved to about 10 mm far from the antenna, the center frequency only shifted from 5.6086 GHz to 5.6076 GHz. It can be noted that when the hand contacted with the antenna in Y-direction and Z-direction (distance in Y-direction or Z-direction is 0 mm), the resonance frequency shift will be more obvious compared with no hand-antenna contact cases, which was caused by the change of effective antenna length by the contact of antenna and other external dielectric materials (hand, in this study). But even in the case of contacting, the resonance frequency shift was still less than 1%. The results indicate that the 3D antenna with a serpentine structure is highly tolerant to the hand effect. In the real application situations of antenna in wearable electronics, the antenna will be attached to a location very close to the human body. The negligible resonance frequency shift of the serpentine-like antenna with the influence of hand indicates that this kind of design can avoid the interference of human body movements when the antenna is used in wearable electronics.

## Conclusion

In this study, a highly stretchable 3D antenna was fabricated by a quick and easy-to-setup “Cut-Transfer-Release” method. The 3D antenna was formed by simply cutting the functional thin foil, transferring the pattern-defined foil to a prestrained substrate and releasing the prestrain. A serpentine structure for the 3D antenna was proposed in this study. The mechanical reliability of the serpentine-like antenna was evaluated by FEM and compared with the conventional ribbon structure. The multiple straight segments of the serpentine structure can effectively reduce the strain concentration on the Au layer and prevent the potential mechanical failures, as indicated by the FEM results. Optical and electrical characterization methods were also used to confirm that the stretchability of the serpentine-like antenna could be as high as 200%, which is suitable for the applications in wearable electronics. The radio frequency performance measurements on the serpentine-like antenna showed robust RF performance in 5.6 GHz band during stretching, indicating the 3D antenna with serpentine structure is promising in the application of 802.11 high-speed wireless communications. The reliable RF performance with high stretchablility can be attributed to the design of transforming the in-plane deformation into out-of-plane deformation, which will lead to only a marginally dimensional change of the serpentine structural unit. Moreover, the measurement results of the S_11_ parameter when the hand is below to, parallel to or on the top of antenna showed the serpentine-like antenna is highly tolerant to the hand effect and the resonance frequency shift caused by the hand effect was less than 1% even when the hand contacted with the antenna. The proposed new “Cut-Transfer-Release” method and the 3D antenna with the serpentine structure are expected to provide a novel way to manufacture low cost antennas for wireless communication of future wearable electronics. The 3D stretchable structure also provides new design directions for a broad range of devices in stretchable electronics, besides the wearable antenna reported in this study.

## Methods

### Finite Element Method Analysis

ABAQUS commercial software was used to study the mechanics response of the 3D serpentine-like antenna on the elastomeric Ecoflex substrate. The Ecoflex substrate was modeled by the hexahedron element (C3D8R), while the Au/PI foil of the 3D serpentine-like layer was modeled by the composite shell element (S4R). An ideal elastic-plastic constitutive relation with a Young’s modulus of 78 GPa, Poisson’s ratio of 0.44 and yield strain of 0.3% describes the mechanical behavior of Au. The Young’s modulus and Poisson’s ratio are 2.5 GPa and 0.34 for PI.

### 3D functional structure fabrication

The elastomeric substrate was made by Ecoflex rubber (Smooth-On, USA, Young’s modulus is 60 kPa), which is a two-component compound requiring thermal curing. The A and B components were first fully mixed by a drill mixer in a 1:1 ratio in weight, followed by being poured into a designed mold with the dimension of 25 mm in length, 25 mm in width and 1.8 mm in height. The mixture of Ecoflex stayed at 27 °C for 24 h. After curing, a Ecoflex elastomeric substrate with expected dimension was obtained. The functional Au thin film layer was deposited by direct current sputtering (50 sccm Ar, 3 Pa, 40 W) of 99.999% Au target on the patterned PI foil for 40 s with a deposition rate of 1.25 nm/s.

### Antenna radio frequency performance measurement

The S_11_ parameters were tested by Agilent Technologies E5071C network analyzer (200 kHz-20 GHz). The calibration of the network analyzer was accomplished using the Agilent 85032F calibration kit. The antenna was connected with the network analyzer by SMA connection.

## Additional Information

**How to cite this article**: Yan, Z. *et al*. Highly stretchable and shape-controllable three-dimensional antenna fabricated by “Cut-Transfer-Release” method. *Sci. Rep.* **7**, 42227; doi: 10.1038/srep42227 (2017).

**Publisher's note:** Springer Nature remains neutral with regard to jurisdictional claims in published maps and institutional affiliations.

## Supplementary Material

Supplementary Information
