# Supplementary material for: Highly stretchable and shape-controllable three-dimensional antenna fabricated by “Cut-Transfer-Release” method
Source: Sci Rep. 2017 Feb 13;7:42227. doi: 10.1038/srep42227 (PMC5304148; doi:10.1038/srep42227)
Supplement: Supplementary Information [file srep42227-s1.doc]

**Supplementary Information**

Highly stretchable and shape-controllable 3D antenna fabricated by “Cut-Transfer-Release” method

Zhuocheng Yan^1,*^, Taisong Pan^1,*^, Guang Yao^1^, Feiyi Liao^1^, Zhenlong Huang^1^, Hulin Zhang^1^, Min Gao^1,2^, Yin Zhang^1^, and Yuan Lin^1,2,a)^

*^1^State Key Laboratory of Electronic Thin Films and Integrated Devices, University of Electronic Science and Technology of China, Chengdu, Sichuan 610054, P. R. China*

*^2^Center for Information in Medicine, University of Electronic Science and Technology of China, Chengdu, Sichuan 610054, P. R. China*

*: Z.C.Y and T.S.P contributed equally to this work.

a): Correspondence and requests for materials should be addressed to Y.L. (email: linyuan@uestc.edu.cn).

**1. The 3D stretchable antenna on the elastomeric substrate**


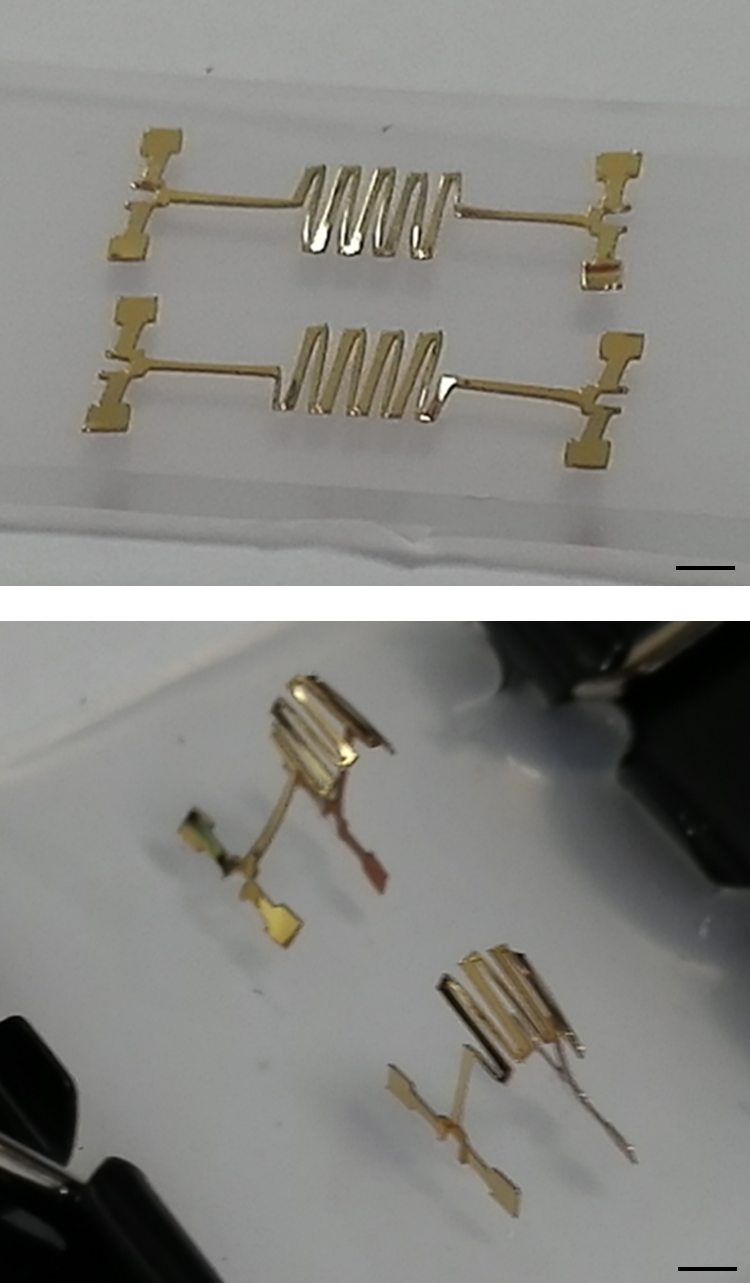


**Figure S1:** The 3D stretchable antenna on the elastomeric substrate with substrate strain 200% (upper panel) and 0 % (lower panel). The scale bar is 1mm.

**2 The formation of 3D antenna from 2D precursor pattern to 3D structures**

**
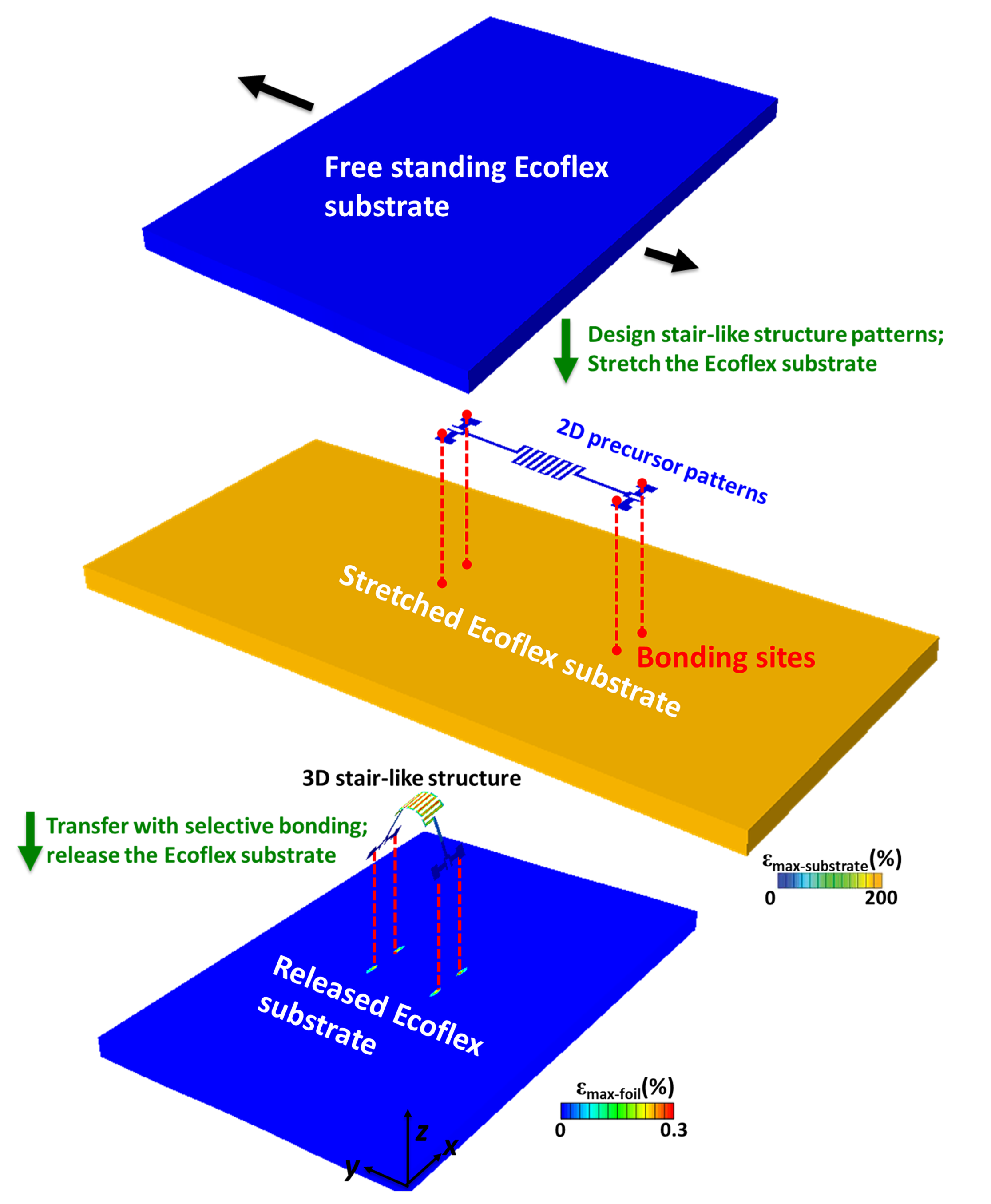
**

**Figure S2:** Finite element method analysis (FEM) results showing the formation of 3D structures from 2D precursor pattern (Au/PI) bonded at selected regions (red dots) to a uniaxial stretched Ecoflex substrate. With the releasing of prestrained Ecoflex substrate, compressive forces lead to the out-of-plane deformations of the 2D Au/PI foil precursor and the 3D functional structure forms.

**3. The detailed size of the 2D precursor pattern of 3D serpentine-like antenna**


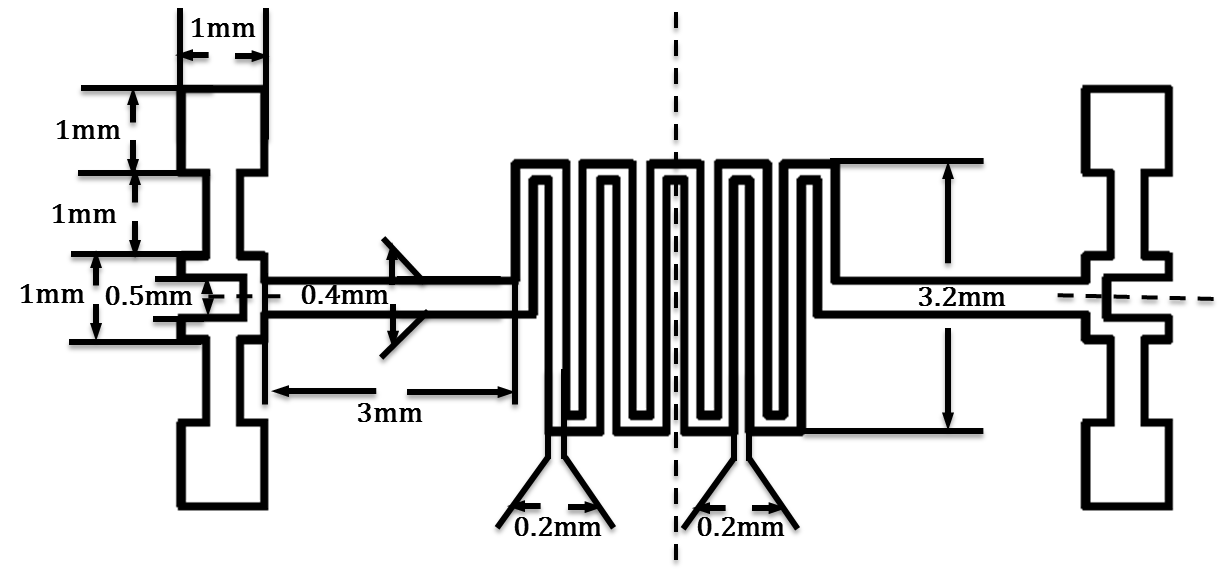


**Figure S3:** The detailed size values of the 2D precursor pattern of 3D serpentine-like antenna

**4. The bonding sites of the 3D 50-nm-thick serpentine-like antenna before and after cyclic stretching**

Figure S4 shows that the bonding sites are well bonded after cyclic stretching

**
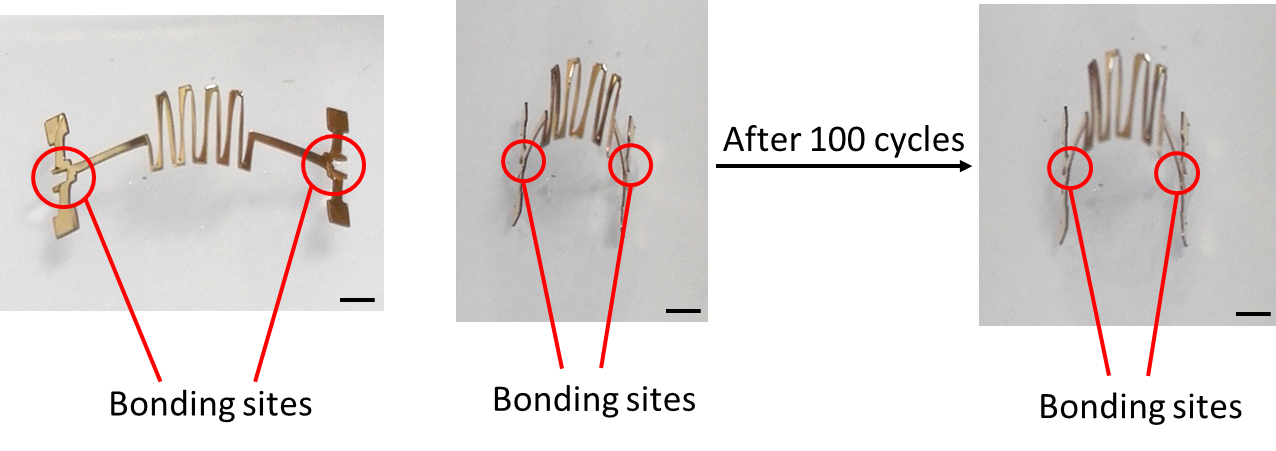
**

**Figure S4:** Optical images of the 3D serpentine-like antenna before and after 100 cycles of stretching with 200% substrate strain (Scale bar 1mm).

**5. The fatigue regions of the 3D 50-nm-thick Au ribbon-like structure after cyclic stretching**

**
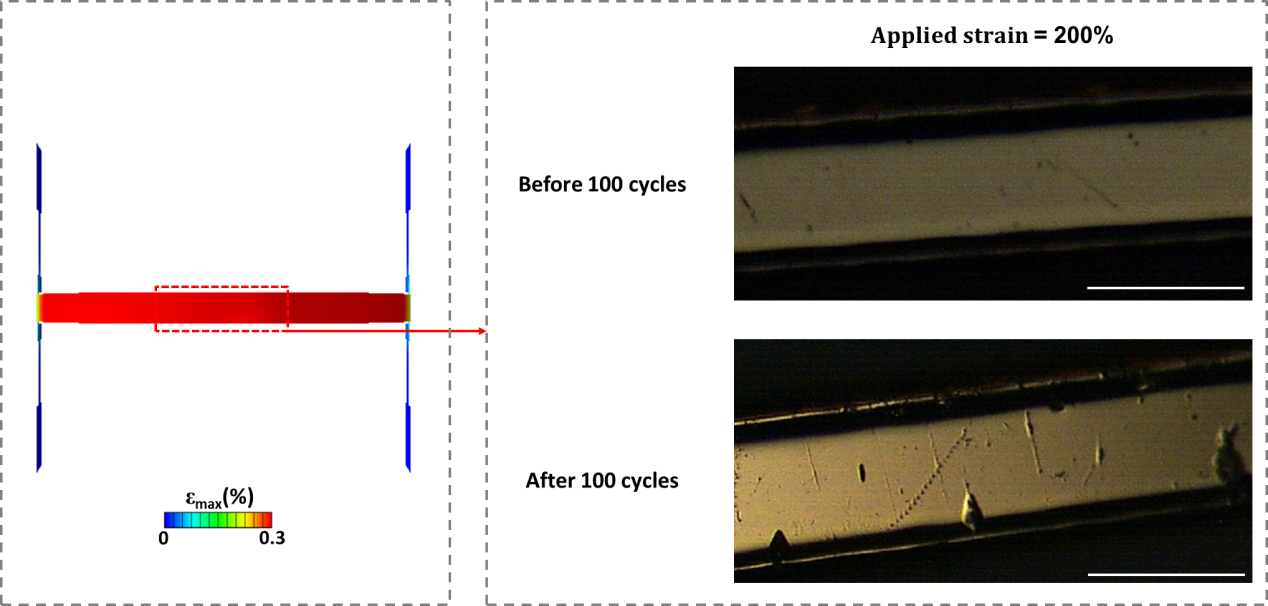
**

**Figure S5:** FEM results for the strain distribution on a 3D 50-nm-thick Au ribbon formed on a released 200% prestrained substrate (left) and optical images of the 3D 50-nm-thick Au ribbon-like structure before and after 100 cycles (right). Fracture regions with cracks can be observed after cyclic stretching with 200% substrate strain. The scale bar is 100 μm.

**6. The resistance change of the 3D Au/PI ribbon-like structure** **after 100 cycles**

**
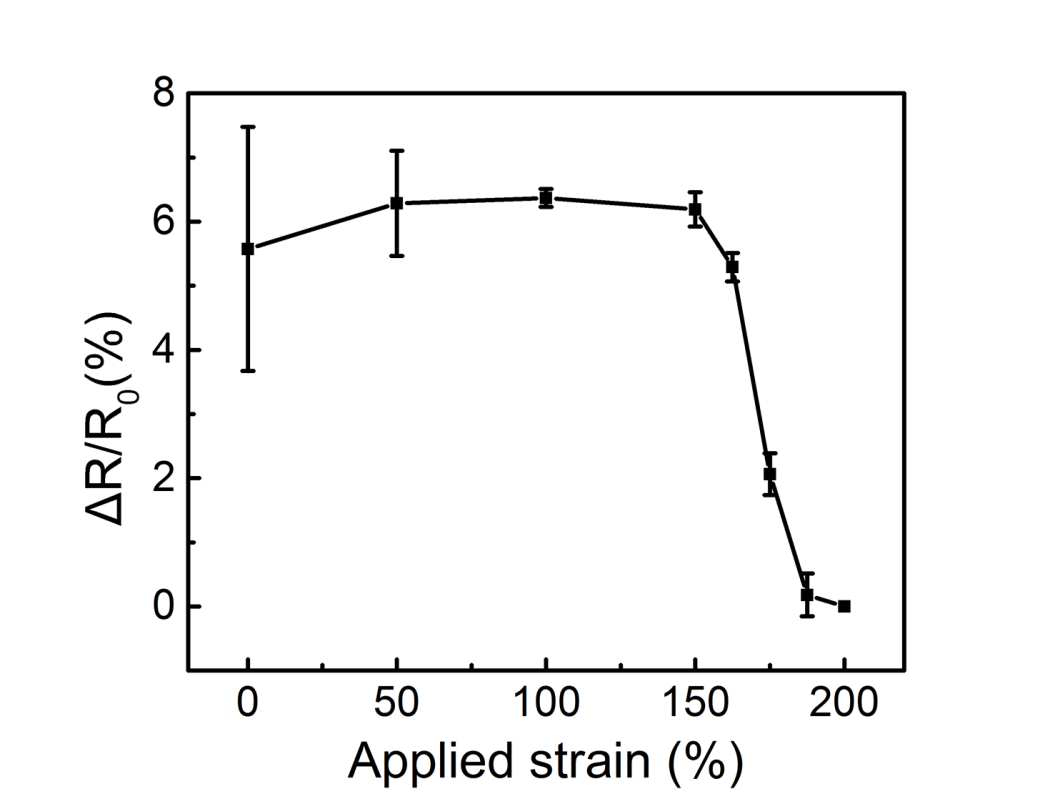
**

**Figure S6:** The resistance change of ribbon-like structure with different substrate strain after stretching to 200% for 100 times (The initial resistance was measured with 200% substrate strain. Change value is average value of 10 samples).

**7.** **The assembly details of 3D serpentine-like antenna in measurement**


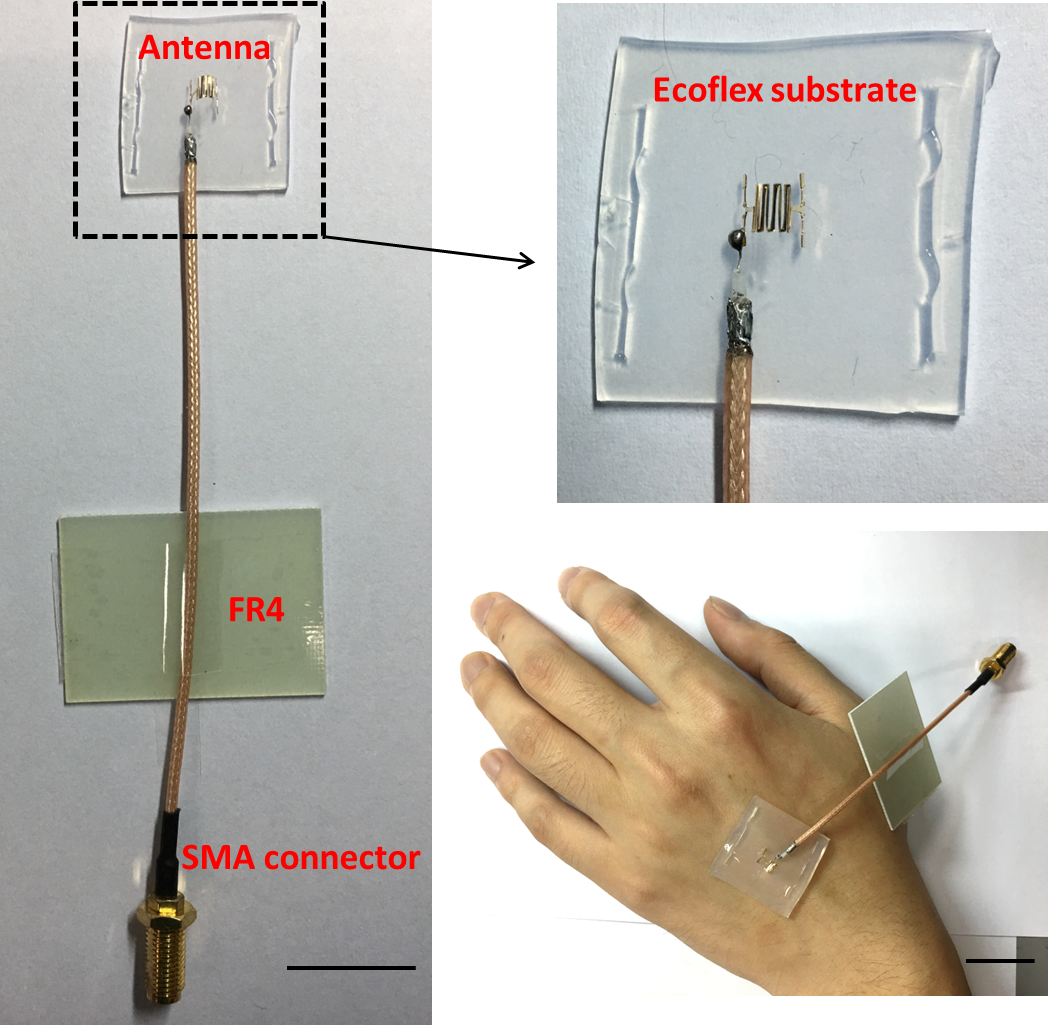


**Figure S7:** Optical image of 3D serpentine-like antenna with SMA connector (left panel) and enlarged image of the antenna (top of right panel). The antenna is mounted on a human hand to conduct the hand effect measurement (bottom of right panel).
